# HNRNPC promotes collagen fiber alignment and immune evasion in breast cancer via activation of the VIRMA-mediated TFAP2A/DDR1 axis

**DOI:** 10.1186/s10020-023-00696-5

**Published:** 2023-08-01

**Authors:** Bin Lian, Shuxun Yan, Jiayi Li, Zhengyang Bai, Jinping Li

**Affiliations:** 1 Department of Surgical Oncology, General Hospital of Ningxia Medical University, No. 804, Shengli Street, Xingqing District, Yinchuan, 750004 Ningxia Hui Autonomous Region China; 2 Ningxia Medical University, Yinchuan, 750004 China; 3 Northwest University for Nationalities, Lanzhou, 730030 China

**Keywords:** Immune evasion, Collagen fiber alignment, DDR1, VIRMA, HNRNPC, TFAP2A, Breast cancer, Collagen remodeling

## Abstract

**Background:**

Cancers aggressively reorganize collagen in their microenvironment, leading to the evasion of tumor cells from immune surveillance. However, the biological significance and molecular mechanism of collagen alignment in breast cancer (BC) have not been well established.

**Methods:**

In this study, BC-related RNA-Seq data were obtained from the TCGA database to analyze the correlation between DDR1 and immune cells. Mouse BC cells EO771 were selected for in vitro validation, and dual-luciferase experiments were conducted to examine the effect of TFAP2A on DDR1 promoter transcription activity. ChIP experiments were performed to assess TFAP2A enrichment on the DDR1 promoter, while Me-RIP experiments were conducted to detect TFAP2A mRNA m6A modification levels, and PAR-CLIP experiments were conducted to determine VIRMA’s binding to TFAP2A mRNA and RIP experiments to investigate HNRNPC’s recognition of m6A modification on TFAP2A mRNA. Additionally, an in vivo mouse BC transplant model and the micro-physiological system was constructed for validation, and Masson staining was used to assess collagen fiber arrangement. Immunohistochemistry was conducted to identify the number of CD8-positive cells in mouse BC tumors and Collagen IV content in ECM, while CD8 + T cell migration experiments were performed to measure CD8 + T cell migration.

**Results:**

Bioinformatics analysis showed that DDR1 was highly expressed in BC and negatively correlated with the proportion of anti-tumor immune cell infiltration. In vitro cell experiments indicated that VIRMA, HNRNPC, TFAP2A, and DDR1 were highly expressed in BC cells. In addition, HNRNPC promoted TFAP2A expression and, therefore, DDR1 transcription by recognizing the m6A modification of TFAP2A mRNA by VIRMA. In vivo animal experiments further confirmed that VIRMA and HNRNPC enhanced the TFAP2A/DDR1 axis, promoting collagen fiber alignment, reducing anti-tumor immune cell infiltration, and promoting immune escape in BC.

**Conclusion:**

This study demonstrated that HNRNPC promoted DDR1 transcription by recognizing VIRMA-unveiled m6A modification of TFAP2A mRNA, which enhanced collagen fiber alignment and ultimately resulted in the reduction of anti-tumor immune cell infiltration and promotion of immune escape in BC.

**Supplementary Information:**

The online version contains supplementary material available at 10.1186/s10020-023-00696-5.

## Background

The interaction between the immune system and malignant cells is essential for tumorigenesis; the immune system’s failure to detect and reject transformed cells is a characteristic hallmark of tumor development, including breast cancer (BC) (Igney AND Krammer 2002; Gil Del Alcazar et al. 2020; Mortezaee 2020). Extracellular matrix (ECM) microstructure and mechanics are crucial to BC growth and invasion into surrounding tissues, and collagen alignment confers significant effects on cancer cell behaviors, with translational potential for anticancer drug development (Huynh et al. 2020). The breast tumor boundary is made of aligned, linear collagen, while the mechanism of the pro-oncogenic impact of aligned collagen remains poorly understood (Brett et al. 2020). Tumors, based on multiple mechanisms, evade the destructive elements of the immune response, thus limiting the efficacy of anti-tumor therapeutic strategies (Vinay et al. 2015). Therefore, it necessitates extensive investigation into the mechanistic understanding related to collagen alignment underlying immune evasion of BC to develop more effective therapies to improve outcomes in BC.

Discoidin domain receptor 1 (DDR1) is a non-integrin collagen tyrosine kinase receptor, which is preferentially expressed in several malignancies and plays a key role in cancer progression and metastasis, especially in BC (Belfiore et al. 2018). DDR1 is significantly increased in BC and inversely correlated with the prognosis of patients; DDR1 stimulates the migration and invasion of BC cells via activation of the Src-FAK signaling (Han et al. 2022). In addition, DDR1 has been demonstrated to instigate immune evasion by promoting collagen fiber alignment (Sun et al. 2021).

An online database at jaspar.genereg.net predicted the presence of transcription factor AP-2 A (TFAP2A) binding sites on the DDR1 promoter region. Overexpression of TFAP2A is capable of stimulating the migration and invasion of BC cells (Xu et al. 2019). TFAP2A is an N(6)-methyladenosine (m6A)-modified mRNA (Meng et al. 2020), and m6A modification has been a well-established mechanism resulting in immune evasion (Lou et al. 2021). m6A is installed by m6A methyltransferases, including VIRMA (Wang et al. 2020b). VIRMA is highly expressed in BC tissues and promotes BC proliferation and metastasis in vivo and in vitro (Qian et al. 2019).

Both heterogeneous nuclear ribonucleoprotein (HNRNPC) and VIRMA, as m6A regulatory factors, have been documented to be correlated to the prognosis of lung adenocarcinoma (Wang et al. 2021). Furthermore, HNRNPC is an m6A regulator gene significantly upregulated in triple-negative breast cancer tumor tissues (Wang et al. 2020a). Meanwhile, HNRNPC is negatively correlated with the infiltration levels of most immune cells in the tumor microenvironment of renal cell carcinoma, especially with CD8^+^ T cells, which are a positive regulator of immunosuppression (Wu et al. 2022). However, the regulatory mechanisms of the HNRNPC/TFAP2A/VIRMA/DDR1 axis contributing to the enhanced immune evasion in BC remain unclear. In the current study, we aim to investigate the underlying mechanism of immune evasion in BC and report a mechanism-driven approach to modulate BC immune evasion.

## Materials and methods

### Obtaining and bioinformatics analysis of breast cancer RNA-Seq data from the TCGA database

Breast cancer-related RNA-Seq data is downloaded from the TCGA database (https://tcga-data.nci.nih.gov/), which includes 1096 breast cancer tissues and 112 normal breast tissues. The ensemble IDs of the samples are converted using the GENCODE Gene Set-09.2019 version annotation file, and differential gene analysis is performed using the T-test. Due to the availability of public data in the TCGA database, ethical approval or informed consent is not required for this study.

CIBERSORT (https://cibersort.stanford.edu/) is used to calculate the relative proportions of immune cells in breast cancer patients in the TCGA dataset, and the correlation between DDR1 expression and immune cells is analyzed using the cor test analysis method. In addition, the upstream transcription factors of DDR1 are predicted using the transcription factor online website (http://bioinfo.life.hust.edu.cn/hTFtarget#!/target).

### Prediction of m6A sites on TFAP2A mRNA

The m6A sites on TFAP2A mRNA were predicted using the cuilab database (http://www.cuilab.cn/sramp). The X-axis represents the localization of m6A sites on mRNA, and the Y-axis represents the predicted scores of m6A sites. The higher the score, the greater the likelihood of m6A modification at that site.

### Construction of overexpression and knockdown lentiviral vectors

The overexpression lentiviral vector pCDH-CMV-MCS-EF1α-copGFP (OE-; System Biosciences, USA, CD511B-1) and the knockdown lentiviral vector pSIH1-H1-copGFP (SH-; System Biosciences, USA, SI501A-1) were purchased to construct lentiviral vectors for overexpression of DDR1 and TFAP2A, and knockdown of DDR1, TFAP2A, VIRMA, and HNRNPC. The lentivirus particles were packaged into HEK-293T cells (iCell-h237, Cellular Biomedicine Group Inc., Shanghai, China) using the lentiviral packaging kit (A35684CN, Invitrogen, USA). The collected supernatant after 48 h was lentivirus with a titer of 1 × 10^8 TU/ml. The lentiviral vector sequences are shown in Supplementary Table 1.

### In vitro culture and grouping intervention of breast cancer cells

Mouse breast cancer cells (i.e., EO771, EMT6, 4T1.2) along with normal mouse mammary epithelial cells (i.e., NMuMG) were purchased from American Type Culture Collection (ATCC) with the catalog numbers CRL-3461, CRL-2755, CRL-3406, and CRL-1636, respectively. All cells were cultivated in DMEM culture medium (SLM-243-B, Sigma-Aldrich, USA) supplemented with 10% fetal bovine serum. According to experimental requirements, EO771 cells were grouped as follows: sh-NC, sh-DDR1, sh-NC + oe-NC, sh-TFAP2A + oe-NC, sh-TFAP2A + oe-DDR1, sh-VIRMA + oe-NC, sh-HNRNPC + oe-NC, sh-VIRMA + oe-TFAP2A, and sh-HNRNPC + oe-TFAP2A. Then, 1 mL of the corresponding lentivirus was mixed with EO771 cells for 8 h. After 24 h, 10 µg/mL puromycin (P8230, Beijing Soleibao Technology Co., Ltd., Beijing, China) was added to screen EO771 cells, and the cells were further cultured for four weeks to establish stably transfected cell lines.

### Western blot

After trypsin digestion, cells from different groups were collected and lysed using RIPA buffer (enhanced version, 89,900, Invitrogen, Car, Cal, USA) containing protease inhibitors. The protein concentration was quantified using a BCA protein assay kit (23,235, Invitrogen, Car, Cal, USA). Subsequently, protein samples were separated by 10% SDS-PAGE gel electrophoresis and transferred onto PVDF membranes. After blocking with 5% BSA for 2 h at room temperature to block non-specific binding, diluted primary antibodies, including DDR1 (CST, 1:1000, #3917), GAPDH (CST, 1:1000, #92,310), VIRMA (Abcam, 1:1000, ab271136), and HNRNPC (Abcam, 1:1000, ab133607), were added and incubated overnight at 4 °C. After washing, the membranes were incubated with HRP-labeled goat anti-rabbit secondary antibodies at room temperature for 1 h. ECL working solution (35,055, Invitrogen, Car, Cal, USA) was then used for membrane exposure for 1 min, and excess ECL reagent was removed. Finally, Western blot images were exposed and analyzed using the Bio-Rad Gel Imaging system and ImageLab software. GAPDH was used as an internal reference, and each experiment was repeated three times.

### RT-qPCR

Total RNA extraction from tissues and cells was done using TRIzol (15,596,026, ThermoFisher, USA), and total RNA concentration and purity were checked using the nanodrop2000 spectrophotometer (ThermoFisher, USA). Per the manufacturer’s instructions, the mRNA was converted to cDNA through reverse transcription using the PrimeScript RT reagent Kit (RR047A, Takara, Japan). PCR was quantified using a 7500 Fast Real-Time PCR System (4,351,106, ThermoFisher, USA). TaKaRa corporation synthesized each gene primer (Supplementary Table 2). The real-time fluorescent quantitative PCR test was run for 40 cycles using the following reaction conditions: 10 min of pre-denaturation at 95℃, a 10-s denaturation at 95℃, a 20-s annealing at 60 ℃, and a 34-s extension at 72 ℃. The internal reference primer used for relative quantitative gene expression analysis was GAPDH. The relative transcription level of the target gene was calculated using the 2-△△CT method, where △△Ct=△Ct of the experimental group - △Ct of the control group, and △Ct = Ct (target gene) - Ct (reference gene). The relative transcription level of the target gene mRNA was calculated as 2-△△Ct. This process was repeated three times for each experiment.

### In vivo tumorigenesis

A mouse xenograft model of breast cancer was established by injecting 2 × 105 cells of stably transferred sh-NC, sh-DDR1, sh-NC + oe-NC, sh-TFAP2A + oe-NC, sh-TFAP2A + oe-DDR1, sh-VIRMA + oe-NC, sh-HNRNPC + oe-NC, sh-VIRMA + oe-TFAP2A, and sh-HNRNPC + oe-TFAP2A into the mammary fat pad of 45 seven-week-old C57BL/6 N mice obtained from the Animal Experimental Center of Ningxia Medical University (Ningxia, China). The mice were accommodated in different cages within an SPF-grade animal laboratory at 60–65% relative humidity and 22–25 ℃ temperature for one week before starting the experiment. The mice’s health status was observed before the experiment. Each group consisted of five randomly chosen mice. The mice were raised in an airlocked SPF-grade animal room after the injection, and the tumor’s growth was observed every three days from the first week onwards. The tumor’s size, measured using a caliper, was recorded as a and b to calculate the tumor volume using the formula π(a2b)/6. The weight of the tumor was measured using a scale. The experiment was repeated three times, and the tumor tissue was divided into two parts to fix with 4% paraformaldehyde or stored in liquid nitrogen for future tissue pathology staining or RNA estimation, respectively. The experiments of the current study were approved by the Animal Ethics Committee of the General Hospital of Ningxia Medical University (No. KYLL-2022-1205).

### Construction of BC micro-physiological system (BC-MPS)

Construct a breast cancer micro-physiological system (BC-MPS) by isolating adipose-derived stromal cells (ASCs) from normal mouse mammary tissue and using them to culture both normal mouse mammary tissue and grouped mouse mammary tumor cells between two layers of ASCs. In brief, ASCs are seeded in a six-well culture plate, with the bottom cell monolayer being cultured on a tissue culture dish and the top cell monolayer being cultured on an UpCell temperature-sensitive culture dish. A 7.5% gelatin solution is solidified on a plunger device and placed on the upper ASCs cell layer for 1.5 h at room temperature, allowing the gel to adhere to the upper ASCs cell layer. The upper cell sheet is then treated in ice-cold water for 1.5 h and released from the UpCell temperature-sensitive culture dish. Normal mouse mammary tissue is washed with PBS, minced, and combined with DMEM culture medium. Mouse mammary tumor cells are implanted in the minced mammary tissue. A 300 µL mixture of mouse mammary tumor cells and mammary tissue is placed on the bottom cell monolayer. A plunger device transfers the upper cell sheet onto the mouse mammary tumor cell-mammary tissue mixture, and the cells are then co-cultured for 14 days.

### Masson’s trichrome staining

Masson’s staining is used to detect the arrangement of collagen fibers in mouse mammary cancer tissue. Tumor tissue paraffin sections are dried in a 65 °C oven for 3 h and subjected to routine dewaxing and dehydration. They are then soaked in 10% trichloroacetic acid and 10% potassium dichromate solution for 40 min each, washed with tap water, and stained with hematoxylin (PT001, purchased from Shanghai Bogu Biological Technology Co., Ltd., Shanghai, China) for 8 min, followed by washing with tap water. They are then soaked in a mixture of 1% Light Green (R21983, purchased from Shangbao Biotech, Shanghai, China) and 1% Ponceau S (R23166, purchased from Shangbao Biotech, Shanghai, China) for 40 min. The reaction is terminated with 1% acetic acid followed by 1% molybdic acid. Finally, a mixture of 1% Brilliant Green and 1% phosphomolybdic acid and tap water is added to stop the reaction. The sections are then routinely dehydrated and sealed with transparent neutral resin. The collagen fibers appear blue-green under a light microscope (BX50; Olympus Corp, Tokyo, Japan).

### Immunohistochemistry

Immunohistochemistry is used to detect the number of CD8-positive cells and the collagen IV content in the ECM of mouse mammary cancer tissue. Mouse tumor tissue is fixed in a 10% formalin solution, dewaxed twice with xylene for 10 min each, and then hydrated with a concentration gradient of 100%, 95%, 75%, and 50% ethanol for 5 min each. One drop of H2O2 is added and incubated at room temperature for 10 min. After adding 0.01 mol/L citrate buffer, the sections are microwave-repaired for 20 min, and a drop of normal goat serum is added. After incubating at room temperature for 5 min, rabbit anti-Collagen IV (1:500, ab6586, Abcam, USA) and rabbit anti-CD8 (1:2000, ab217344, Abcam, USA) primary antibodies are added and incubated overnight at 4 °C. The sections are then incubated at 37 °C for 1 h with biotinylated goat anti-rabbit secondary antibodies (1:500, ab150077, Abcam, USA) and incubated at 37 °C for 30 min. Freshly prepared DAB (DA1015, Beijing Sulibao Technology Co., Ltd., Beijing, China) chromogenic solution is added and allowed to react for 1–2 min. The sections are then counterstained with hematoxylin (G1080, Beijing Sulibao Technology Co., Ltd., Beijing, China) for 1 min, dehydrated, and sealed with neutral resin. Five representative high-power fields are randomly selected and observed under a light microscope (BX50; Olympus Corp, Tokyo, Japan).

### CD8 + T cell migration assay

MagniSort™ Mouse CD8 + T Cell Enrichment Kit (8804-6822-74, ThermoFisher, USA) was used to isolate CD8 + T cells from breast cancer mouse tumor tissue. The tumor tissue from the orthotopic breast cancer mouse model was treated to obtain tumor extracellular matrix (ECM), and then ECM and CD8 + T cells (5 × 105 cells) were placed in the upper chamber. CCL21 chemokine (7338–50, Wuhan Aimijie Technology Co., Ltd., Wuhan) was added to the lower chamber tumor-conditioned medium, and the cells were moved for 2 h at 37 °C. Flow cytometry was used to detect the number of CD8 + T cells that migrated to the lower chamber, and the number of CD8 + T migration cells without ECM and CCL12 was set as “1”.

### Dual-luciferase reporter assay

The transcription factor TFAP2A was predicted to have a binding site with the DDR1 promoter region through the Jaspar database (jaspar.genereg.net). Lipofectamine 2000 reagent kit (11,668,019, ThermoFisher, USA) was used to co-transfect oe-NC, oe-TFAP2A, sh-NC, and sh-TFAP2A with a luciferase reporter plasmid containing the DDR1 promoter sequence (GCCGGGAGC) into human embryonic kidney HEK293T cells. Firefly luciferase was used as a control, and 48 h after transfection, the cells were collected and lysed. The luciferase activity was detected using a Luciferase Assay Kit (K801-200, Biovision Corp., USA), and the luciferase reporter gene was analyzed using the Dual-Luciferase Reporter Assay System (Promega, Madison, WI, USA). The activation level of the target reporter gene was compared based on the ratio of the firefly luciferase measurement value (RLU) to the sea kidney luciferase measurement value (RLU).

### ChIP assay

A ChIP assay kit (KT101-02, Saicheng Biotech Co., Ltd., Guangzhou, China) was used to detect the enrichment of TFAP2A on the DDR1 gene promoter region. The procedure was as follows: when the cell fusion rate reached 70–80%, 1% formaldehyde was added to cross-link the DNA and protein in the cells at room temperature for 10 min. After cross-linking, the cells were randomly broken by ultrasonic treatment, with 10-second intervals and 10-second breaks, repeated 15 times, to obtain fragments of appropriate size. The cells were centrifuged at 13,000 rpm at 4 °C. The supernatant was divided into three tubes, each added with positive control antibody RNA Polymerase II Rabbit antibody (1:100, ab238146, Abcam, UK), negative control antibody rabbit IgG (1:100, ab172730, Abcam, UK), and specific protein antibody mouse anti-TFAP2A (1:250, MA1-872, ThermoFisher, USA). After overnight incubation at 4 °C, the Protein Agarose/Sepharose precipitated endogenous DNA-protein complexes. After brief centrifugation, the supernatant was discarded, and non-specific complexes were washed. The cross-linking was reversed overnight at 65 °C, and the DNA fragments were extracted with phenol/chloroform and purified to recover the DDR1 gene promoter fragment, which was detected using qPCR. The DDR1 gene promoter-specific primers were as follows: Forward: 5’-TGGGGAGTGGGTTCCATTAC-3’; Reverse: 5’-TGCCACCTCCCAAGTCATTC-3’.

### Me-RIP assay

Total RNA was isolated from EO771 cells using the Trizol method, and mRNA was purified from total RNA using PolyATtract® mRNA Isolation Systems (61,006, ThermoFisher, USA). For experimental grouping, anti-m6A antibody (1:500, ab151230, Abcam, Cambridge, UK) or anti-IgG antibody (ab109489, 1:100, Abcam, Cambridge, UK) were added to IP buffer (20-mM Tris pH 7.5, 140-mM NaCl, 1% NP-40, 2-mM EDTA) and incubated with protein A/G magnetic beads for 1 h to allow binding. Next, the purified mRNA and magnetic bead-antibody complex were added to an IP buffer containing ribonuclease and protease inhibitors and incubated overnight at 4 °C. Finally, the RNA was eluted using elution buffer, purified using phenol-chloroform extraction, and analyzed using RT-qPCR to detect the expression level of TFAP2A mRNA. The experiment was repeated three times.

### PAR-CLIP assay

EO771 cells were incubated with 4-thiouridine (148,202, Sigma-Aldrich, USA) for 14 h and cross-linked at 365 nm with 0.4 J/cm2. After lysis, immunoprecipitation was performed using VIRMA antibody (NBP1-21364, 1:200, Novusbio, USA), and radiolabeled RNA was precipitated using [g-32-P]-ATP. PAR-fragment RT-qPCR analysis was carried out by removing proteins using proteinase K digestion and extracting RNA to detect the expression level of TFAP2A mRNA.

### RIP assay

RIP assay was performed using RIP kit (KT102-01, SaiCheng BioTech, Guangzhou, China) with HNRNPC as the antibody to detect the recognition of HNRNPC to m6A modification of TFAP2A mRNA. After washing with cold PBS, the cells under test were lysed with RIPA lysis buffer (P0013C, Bi Yun Tian, Shanghai, China) on ice for 5 min, and the supernatant was collected by centrifugation at 4 °C for 10 min. Then, the cell lysate was incubated with the antibody to co-precipitate, and the magnetic bead-antibody complex was collected. RNA was extracted after protein digestion with proteinase K to further detect TFAP2A. The antibody used for RIP was HNRNPC (GTX113463, 1:200, Genetex, USA), and IgG (ab172730, 1:100, Abcam, UK) was used as a negative control.

### Statistical analysis

The statistical analysis of this study data was performed using SPSS software (version 21.0, IBM, USA). The data were expressed as Mean ± SD. Unpaired Student’s t-test was used to compare two normally distributed data, and one-way ANOVA was used to compare data among multiple groups with Tukey’s post-hoc test. P < 0.05 indicated a statistically significant difference.

## Results

### DDR1 is highly expressed in BC and inversely correlates to the ratio of anti-tumor immune cell infiltration

**Fig. 1:**
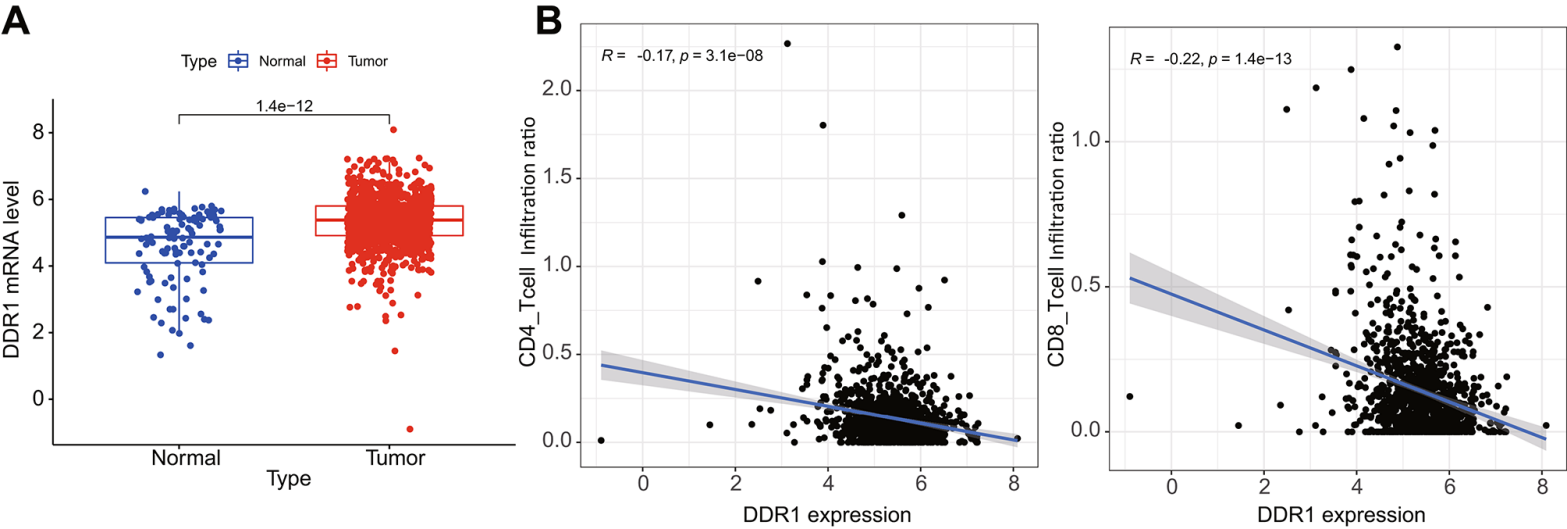
Expression of DDR1 and its correlation with immune cells in breast cancer. Note: (**A**) The expression levels of DDR1 in breast cancer-related TCGA dataset, including 112 normal cases and 1096 tumor cases; (**B**) Correlation analysis of DDR1 and the proportion of anti-tumor immune cell infiltration in breast cancer-related TCGA dataset

DDR1 is a receptor tyrosine kinases (RTKs) family member, with collagen as its ligand. As a novel RTK protein, DDR1 has become one of the potential targets for cancer treatment (Valiathan et al. 2012). In order to investigate the mechanism of DDR1 in breast cancer, we obtained breast cancer-related TCGA data. The results showed that DDR1 was highly expressed in breast cancer tissues (Fig. 1A). A previous study has indicated that the expression of DDR1 helps to establish a physical barrier around the tumor, preventing T cells from infiltrating into the tumor and hindering their ability to kill tumor cells (Zhang et al. 2022). Immune correlation analysis of breast cancer-related TCGA data revealed that the expression of DDR1 was negatively correlated with the ratio of anti-tumor immune cells CD8 + T cells and CD4 + T cells infiltrating the tumor (Fig. 1B).

### DDR1 promotes immune evasion of BC by enhancing collagen IV synthesis and collagen remodeling in the ECM of BC

**Fig. 2:**
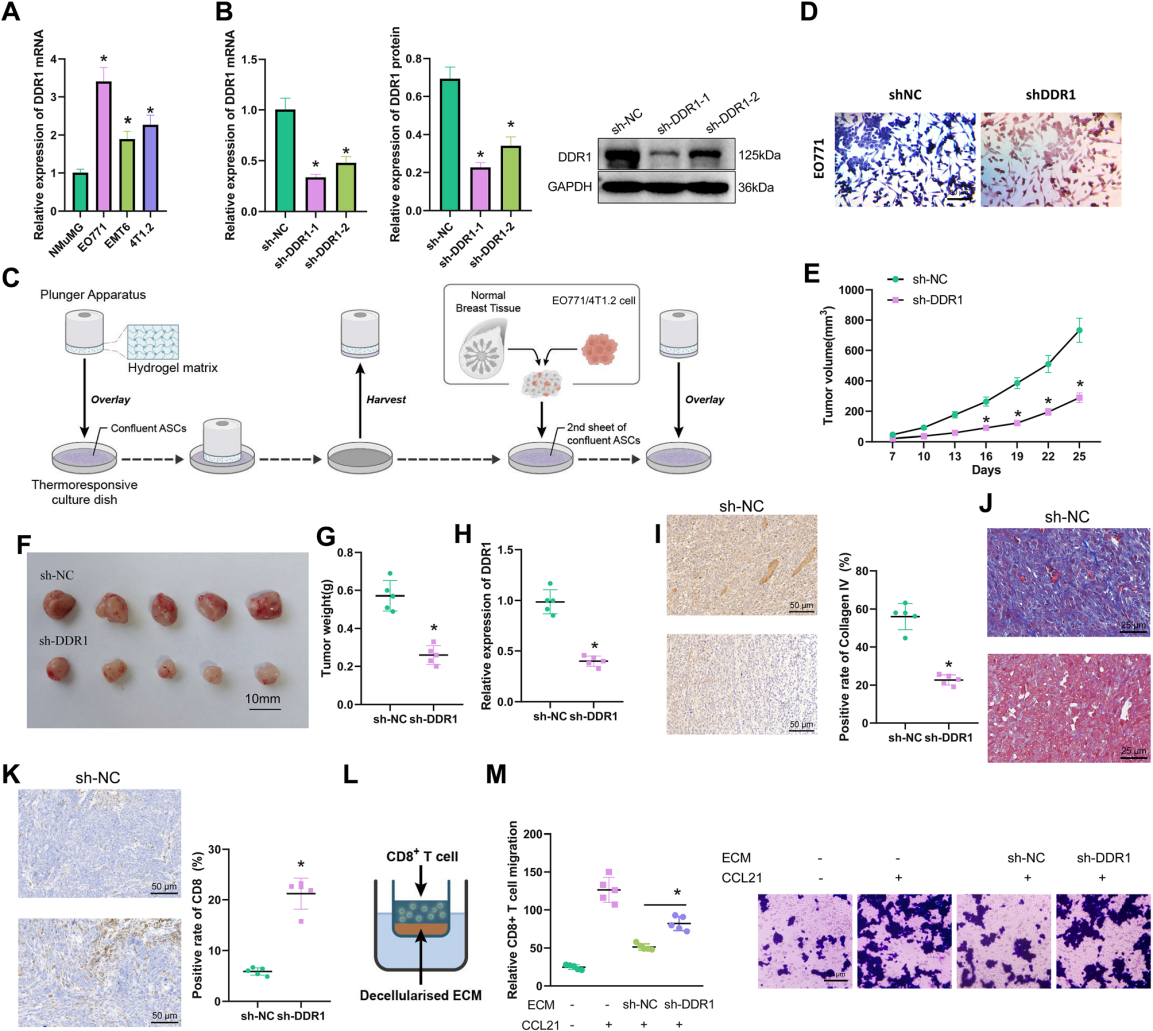
Effects of DDR1 on collagen fiber arrangement and immune escape in breast cancer. Note: (**A**) RT-qPCR detected the expression levels of DDR1 in breast cancer cell lines (EO771, EMT6, and 4T1.2), * indicates P < 0.05, ** indicates P < 0.01 compared with the NMuMG cells; (**B**) RT-qPCR and Western blot detected the silencing efficiency of DDR1 in EO771 cells, * indicates P < 0.05, ** indicates P < 0.01 compared with the sh-NC group; (**C**) Schematic diagram of BC-MPS construction; (**D**) Masson staining detected the collagen fiber arrangement in each group of BC-MPS (×100); (**E**) Tumor volume of each group of breast cancer mice; (**F**) Photos of tumors taken from each group of mice; (**G**) Tumor weight of each group of breast cancer mice; (**H**) RT-qPCR detected the expression levels of DDR1 in each group of breast cancer mice tumor tissues; (**I**) Immunohistochemistry detected the content of Collagen IV in ECM of each group of breast cancer mice tumor tissues (×200); (**J**) Masson staining detected the collagen fiber arrangement in each group of breast cancer mice tumor tissues (×400); (**K**) Immunohistochemistry detected the number of CD8-positive cells in each group of breast cancer mice tumor tissues (×200); (**L**) Schematic diagram of CD8 + T cell migration experiment; (**M**) CD8 + T cell migration experiment detected the migration of CD8 + T cells in each group of breast cancer mice tumor tissues after decellularized ECM treatment. * indicates P < 0.05, ** indicates P < 0.01 compared with the control group. All cell experiments were repeated 3 times; each group consisted of 5 mice

The literature reports that DDR1 can strengthen the arrangement of collagen fibers by binding to collagen proteins in the extracellular matrix (ECM), thereby inhibiting immune infiltration (Sun et al. 2021). To explore the role of DDR1 in breast cancer, we first used RT-qPCR to detect the expression levels of DDR1 in breast cancer cells. The results showed (Fig. 2A) that compared with normal breast epithelial cells NMuMG, the expression of DDR1 in various breast cancer cell lines was significantly increased, and the changes in DDR1 expression were most significant in EO771 cells and 4T1.2 cells. Therefore, EO771 and 4T1.2 cell lines were selected for subsequent experiments. Subsequently, we silenced DDR1 in EO771 cells, and the RT-qPCR and Western blot results showed (Fig. 2B) that the expression of DDR1 was significantly reduced after DDR1 silencing, with better silencing effects observed in sh-DDR1-1. Furthermore, the RT-qPCR and Western blot results for 4T1.2 cells also showed better silencing effects of sh-DDR1-1 (Fig. S1A). Hence we chose sh-DDR1-1 for subsequent experiments.

Next, we constructed a breast cancer micro physiological system (BC-MPS) by culturing normal breast tissue and breast cancer cells that had been treated into groups between two adipose-derived stromal cells (ASCs) (Fig. 2C). Masson staining was used to detect the arrangement of collagen fibers in the BC-MPS. The results showed (Fig. 2D, Fig. S1B) that after silencing DDR1 in EO771 and 4T1.2 cells, the collagen fibers in the BC-MPS were sparsely distributed. Since the results in Fig. 2A showed that the expression of DDR1 was significant in EO771 cells, we chose to inject the E0771 cells into the mammary fat pad of mice to construct a breast cancer mouse orthotopic transplantation model and subjected them to group treatments. The statistical results of tumor volume and weight showed (Fig. 2E, F, G) that after silencing DDR1, both the tumor volume and weight decreased significantly. In addition, RT-qPCR results showed (Fig. 2H) that after silencing DDR1, the expression of DDR1 in the tumor tissue was significantly reduced.

Collagen IV is a basement membrane protein, and destroying this extracellular matrix structure is an important process for cancer invasion (Jansson et al. 2022). Immunohistochemistry and Masson staining results showed (Fig. 2I-J) that after silencing DDR1, the content of Collagen IV in the tumor ECM was significantly reduced, and the distribution of collagen fibers was sparsely arranged. In addition, Immunohistochemistry was used to detect CD8-positive cells in tumor tissue, and the results showed (Fig. 2K) that after silencing DDR1, the number of CD8-positive cells in tumor tissue increased significantly, and CD8-positive cells could be observed migrating from the tumor stroma to the tumor parenchyma. These results indicate that silencing DDR1 can reduce the content of Collagen IV in the ECM, disrupt the arrangement of collagen fibers, increase immune cell infiltration, and inhibit tumor growth.

Furthermore, tumor extracellular matrix sh-NC (ECM) and sh-DDR1 (ECM) were obtained by removing cells from the tumor tissue in the two groups of breast cancer mouse orthotopic transplantation models. Subsequently, ECM and CD8 + T cells were placed in the upper chamber, and the lower chamber was added with the chemokine CCL21 for CD8 + T cell migration experiments (Fig. 2L). The results showed (Fig. 2M) that after silencing DDR1, the number of CD8 + T cell migrations increased significantly. It indicates that silencing DDR1 can increase tumor CD8 + T cell infiltration by reducing the content of Collagen IV in the ECM and disrupting the arrangement of collagen fibers.

### Transcription factor TFAP2A promotes DDR1 transcription by binding to the DDR1 promoter region

**Fig. 3:**
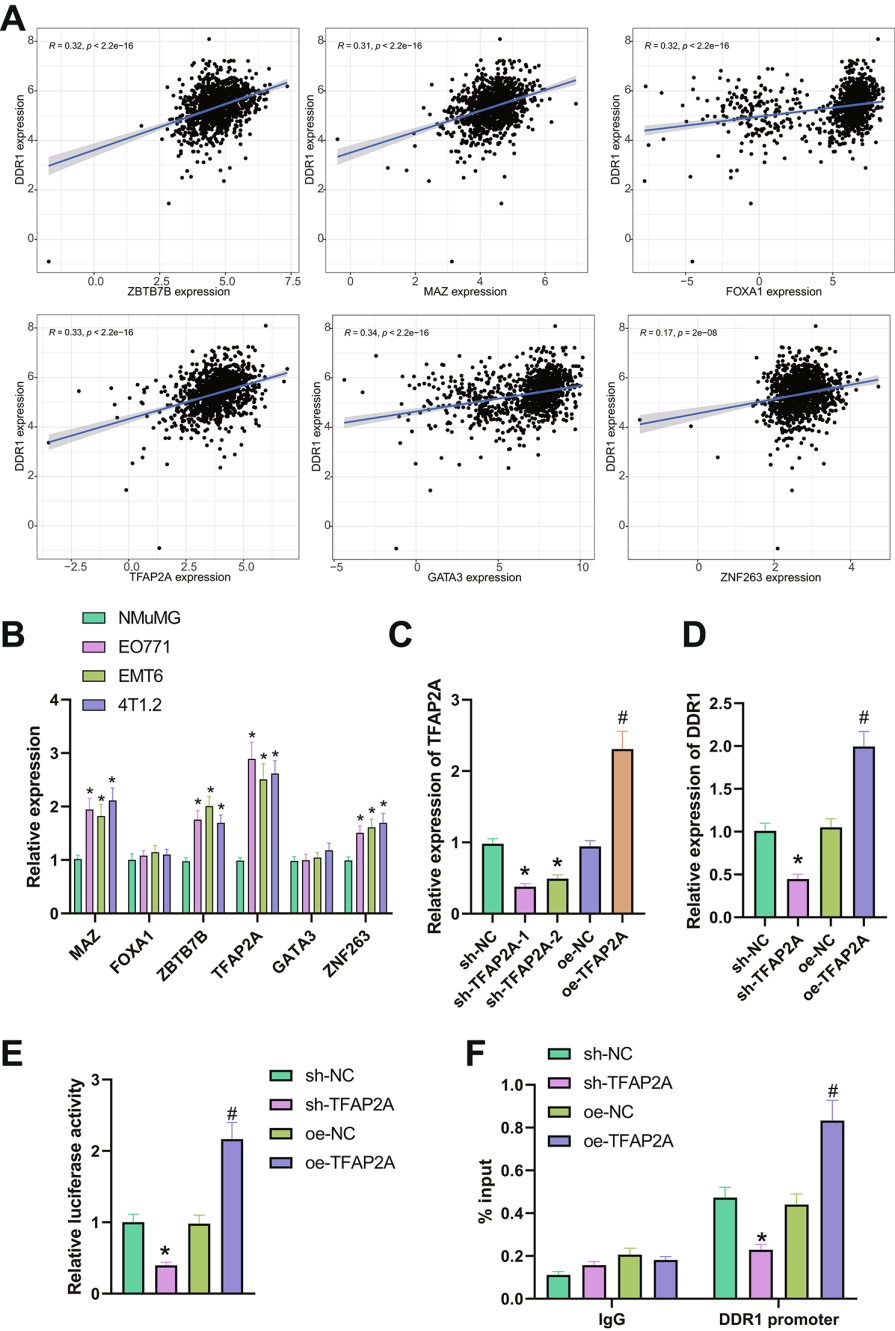
Regulation of DDR1 by transcription factor TFAP2A. Note: (**A**) Correlation analysis of DDR1 and transcription factors MAZ, FOXA1, ZBTB7B, TFAP2A, GATA3, and ZNF263 in breast cancer-related TCGA dataset, including 1096 breast cancer tissues; (**B**) RT-qPCR detected the expression levels of MAZ, FOXA1, ZBTB7B, TFAP2A, GATA3, and ZNF263 in breast cancer cells (EO771, EMT6, and 4T1.2); (**C**) RT-qPCR detected the expression levels of TFAP2A in each group of EO771 cells after silencing or overexpression; (**D**) RT-qPCR detected the expression levels of DDR1 in each group of EO771 cells after silencing or overexpression; (**E**) Dual-luciferase assay detected the activity of DDR1 promoter in each group of EO771 cells after silencing or overexpression of TFAP2A; (**F**) ChIP assay detected the enrichment of TFAP2A on DDR1 promoter in each group of EO771 cells after silencing or overexpression of TFAP2A. * indicates P < 0.05, ** indicates P < 0.01 compared with the control group. All cell experiments were repeated 3 times

Currently, the molecular regulatory mechanism of DDR1 in breast cancer is not clear, but it is known that transcription factors are widely involved in the progression of breast cancer (Lei et al. 2022). To explore whether DDR1 can be regulated through the transcription factor pathway, we first predicted the upstream transcription factors of DDR1 through a transcription factor online website, and combined with the breast cancer-related TCGA dataset, we screened for transcription factors that are significantly correlated with the expression of DDR1, including MAZ, FOXA1, ZBTB7B, TFAP2A, GATA3, and ZNF263 (Fig. 3A). The RT-qPCR results showed that compared with normal breast epithelial cells NMuMG, the expression of FOXA1 and GATA3 in breast cancer cells did not change significantly, while the expression of MAZ, ZBTB7B, TFAP2A, and ZNF263 significantly increased, of which TFAP2A showed the most significant increase (Fig. 3B). There is currently no related report on the regulatory relationship between TFAP2A and DDR1, so we selected it as the research object.

Next, we grouped and treated the EO771 cells and 4T1.2 cells. The RT-qPCR results showed (Fig. 3C, Fig. S1C) that after silencing TFAP2A, the expression of TFAP2A decreased significantly, among which the silencing effect of sh-TFAP2A-1 was better, so we selected it for subsequent experiments; after overexpressing TFAP2A, the expression of TFAP2A significantly increased. Further RT-qPCR detection found (Fig. 3D, Fig. S1D) that after silencing TFAP2A, the expression of DDR1 in EO771 cells and 4T1.2 cells both significantly decreased; after overexpressing TFAP2A, the expression of DDR1 significantly increased. It indicates that TFAP2A can regulate the expression of DDR1. Next, we cloned the promoter region of DDR1 to the upstream of the luciferase gene (Fig. S1E). After different treatment groups (sh-NC, shTFAP2A, oe-NC, oeTFAP2A), we detected the strength of luciferase and evaluated the strength of DDR1 promoter activity. The results of the dual luciferase experiment showed (Fig. 3E, Fig. S1F) that after silencing TFAP2A, the activity of the DDR1 promoter significantly decreased, and after overexpressing TFAP2A, the activity of the DDR1 promoter significantly increased. The results of the ChIP experiment showed (Fig. 3F, Fig. S1G) that after silencing TFAP2A, the enrichment of TFAP2A on the DDR1 promoter significantly decreased. However, after overexpressing TFAP2A, the enrichment of TFAP2A on the DDR1 promoter significantly increased.

### TFAP2A reduces BC immune cell infiltration by elevating DDR1 expression and enhancing collagen fiber alignment

**Fig. 4:**
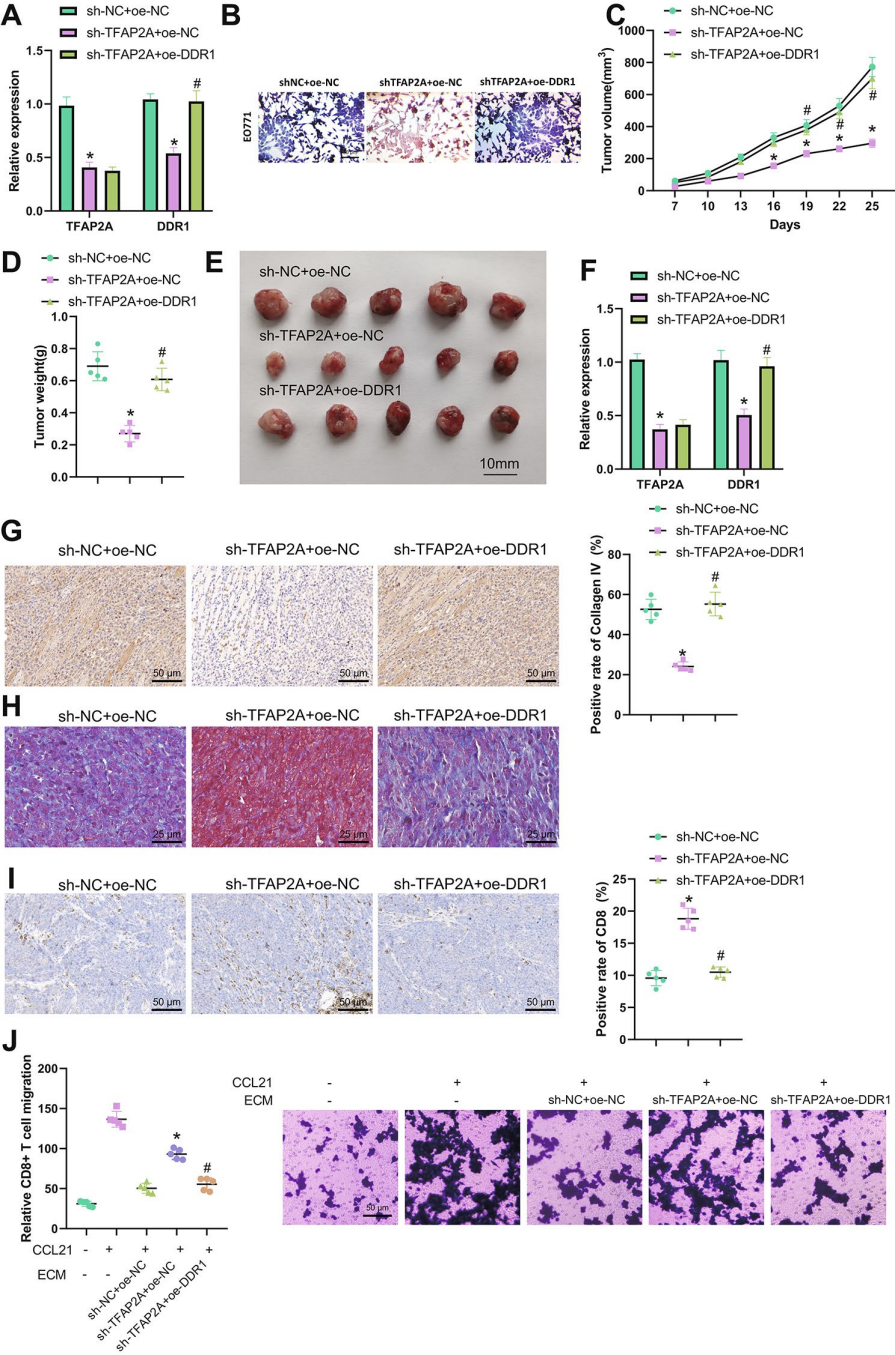
Effects of TFAP2A-mediated DDR1 regulation on collagen fiber arrangement and immune cell infiltration in breast cancer. Note: (**A**) RT-qPCR detected the expression levels of TFAP2A and DDR1 in each group of EO771 cells; (**B**) Masson staining detected the collagen fiber arrangement in each group of BC-MPS treated with different EO771 cells (×100); (**C**) Tumor volume of each group of breast cancer mice; (**D**) Tumor weight of each group of breast cancer mice; (**E**) Photos of tumors taken from each group of mice; (**F**) RT-qPCR detected the expression levels of TFAP2A and DDR1 in each group of breast cancer mice tumor tissues; (**G**) Immunohistochemistry detected the content of Collagen IV in ECM of each group of breast cancer mice tumor tissues (×200); (**H**) Masson staining detected the collagen fiber arrangement in each group of breast cancer mice tumor tissues (×400); (**I**) Immunohistochemistry detected the number of CD8-positive cells in each group of breast cancer mice tumor tissues (×200); (**J**) CD8 + T cell migration experiment detected the migration of CD8 + T cells in each group of breast cancer mice tumor tissues after decellularized ECM treatment. * indicates P < 0.05 compared with the control group. All cell experiments were repeated 3 times; each group consisted of 5 mice

In order to further explore the effect of TFAP2A on breast cancer immune escape through the regulation of DDR1, we grouped and treated EO771 cells and 4T1.2 cells. The RT-qPCR results showed (Fig. 4A, Fig. S2A): Compared with the sh-NC + oe-NC group, the expression of TFAP2A and DDR1 in sh-TFAP2A + oe-NC group cells was significantly reduced; compared with the sh-TFAP2A + oe-NC group, the expression of DDR1 in sh-TFAP2A + oe-DDR1 group cells was significantly increased, and TFAP2A expression was not significantly changed. Next, BC-MPS were constructed according to the cell grouping. Masson staining results showed (Fig. 4B, Fig. S2B): that compared with the sh-NC + oe-NC group, the collagen fiber distribution in the BC-MPS of the sh-TFAP2A + oe-NC group was sparse; compared with the sh-TFAP2A + oe-NC group, the collagen fibers in the BC-MPS of the sh-TFAP2A + oe-DDR1 group showed a high level of directional distribution.

In the in vivo experiment, EO771 transplanted breast cancer mice were grouped and treated. The statistics of tumor volume and weight showed (Fig. 4C-E): Compared with the sh-NC + oe-NC group, the tumor volume and weight of the sh-TFAP2A + oe-NC group were significantly reduced; compared with the sh-TFAP2A + oe-NC group, the tumor volume and weight of the sh-TFAP2A + oe-DDR1 group were significantly increased. Furthermore, the RT-qPCR results showed (Fig. 4F): Compared with the sh-NC + oe-NC group, the expression of TFAP2A and DDR1 in the tumor tissues of the sh-TFAP2A + oe-NC group was significantly reduced; compared with the sh-TFAP2A + oe-NC group, the expression of DDR1 in the tumor tissues of the sh-TFAP2A + oe-DDR1 group was significantly increased, and TFAP2A expression was not significantly changed.

Immunohistochemistry and Masson staining results showed (Fig. 4G-H): compared with the sh-NC + oe-NC group, the content of Collagen IV in the tumor ECM of the sh-TFAP2A + oe-NC group was significantly reduced, and the distribution of collagen fibers was sparse; compared with the sh-TFAP2A + oe-NC group, the content of Collagen IV in the tumor ECM of the sh-TFAP2A + oe-DDR1 group was significantly increased, and the distribution of collagen fibers showed a high level of directional distribution. Immunohistochemistry results showed (Fig. 4I): Compared with the sh-NC + oe-NC group, the number of CD8-positive cells in the tumor tissues of the sh-TFAP2A + oe-NC group was significantly increased; compared with the sh-TFAP2A + oe-NC group, the number of CD8-positive cells in the tumor tissues of the sh-TFAP2A + oe-DDR1 group was significantly reduced. This result indicates that TFAP2A can promote the expression of DDR1 to increase the content of Collagen IV in the ECM, strengthen the arrangement of collagen fibers, reduce the infiltration of breast cancer immune cells, and promote tumor growth.

The results of CD8 + T cell migration experiments (Fig. 4J) showed: compared with the sh-NC + oe-NC group, the number of CD8 + T cell migrations in the sh-TFAP2A + oe-NC group was significantly increased; compared with the sh-TFAP2A + oe-NC group, the number of CD8 + T cell migration in the sh-TFAP2A + oe-DDR1 group was significantly reduced. It indicates that TFAP2A can reduce the infiltration of tumor CD8 + T cells by promoting the expression of DDR1 to increase the content of Collagen IV in the ECM and strengthen the arrangement of collagen fibers.

### HNRNPC augments TFAP2A expression by recognizing the m6A modification of TFAP2A mRNA by VIRMA

**Fig. 5:**
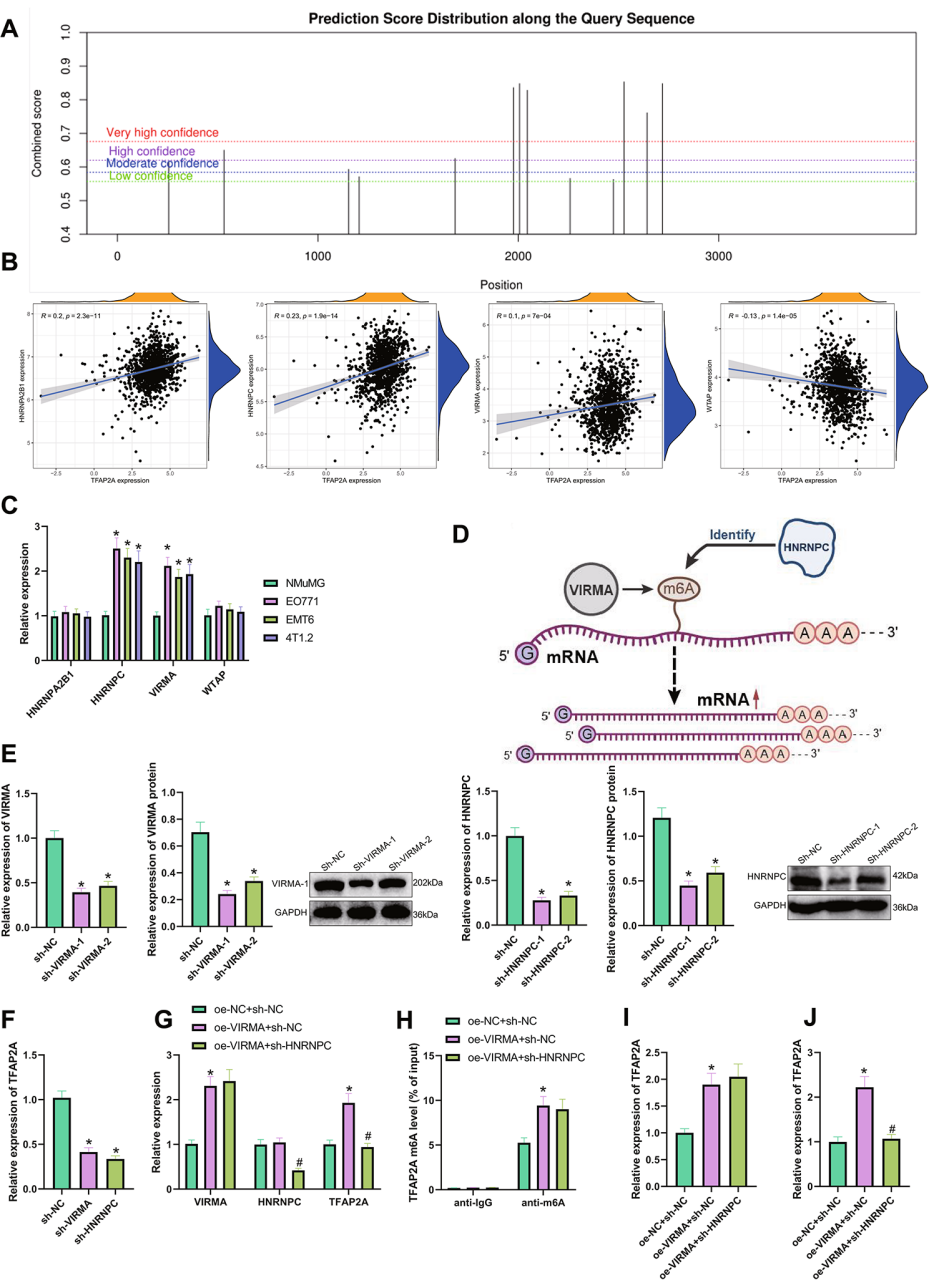
VIRMA and HNRNPC participate in the m6A regulation of TFAP2A. Note: (**A**) Cuilab database predicted the m6A sites on TFAP2A mRNA, and the abscissa represents the location of the m6A sites on mRNA, and the ordinate represents the prediction score of the m6A sites, and the higher the score, the greater the possibility of m6A modification at the site; (**B**) Correlation analysis of TFAP2A and m6A regulatory factors HNRNPA2B1, HNRNPC, VIRMA, and WTAP in breast cancer-related TCGA dataset, including 1096 breast cancer tissues; (**C**) RT-qPCR detected the expression levels of HNRNPA2B1, HNRNPC, VIRMA, and WTAP in breast cancer cells (EO771, EMT6, and 4T1.2); (**D**) Schematic diagram of m6A regulatory mechanism; (**E**) RT-qPCR and Western blot detected the expression levels of VIRMA and HNRNPC in each group of EO771 cells after silencing; (**F**) RT-qPCR detected the expression levels of TFAP2A in each group of EO771 cells after silencing of VIRMA and HNRNPC; (**G**) RT-qPCR detected the expression levels of VIRMA, HNRNPC, and TFAP2A in each group of EO771 cells; (**H**) Me-RIP detected the m6A methylation level of TFAP2A mRNA in each group of EO771 cells; (**I**) PAR-CLIP experiment detected the binding of VIRMA to TFAP2A mRNA in each group of EO771 cells; (**J**) RIP experiment detected the m6A modification recognition of TFAP2A mRNA by HNRNPC in EO771 cells. * indicates P < 0.05, ** indicates P < 0.01 compared with the control group. All cell experiments were repeated 3 times

m6A is considered a key regulatory factor for tumors and the immune system and plays an important role in tumor growth and immune evasion (Li et al. 2022). Therefore, to further explore the molecular mechanism of DDR1 involved in breast cancer immune evasion, we predicted the m6A site on TFAP2A mRNA through the Scilab database. As a result, we found (Fig. 5A): There are 13 m6A sites on TFAP2A mRNA, indicating that TFAP2A mRNA can undergo m6A methylation.

Subsequently, we extracted m6A methylation regulatory factors from the breast cancer-related TCGA dataset and performed a correlation analysis with TFAP2A expression. The results showed (Fig. 5B) that HNRNPA2B1, HNRNPC, VIRMA, and WTAP were significantly correlated with TFAP2A expression. RT-qPCR results (Fig. 5C) showed no significant changes in HNRNPA2B1 and WTAP expression in breast cancer cells compared to normal breast epithelial cells NMuMG. However, VIRMA and HNRNPC expression was significantly increased. As shown in Fig. 5D, VIRMA can act as an m6A methyltransferase (m6A Writer) to promote m6A methylation modification of gene mRNA (Jiang et al. 2021), while HNRNPC, as an m6A recognition factor (m6A Reader), can promote gene expression by recognizing the m6A modification.

Next, we divided EO771 and 4T1.2 cells into groups. RT-qPCR and western blot results showed (Fig. 5E, Fig. S2C) that the expression of VIRMA and HNRNPC was significantly decreased after their knockdown, among which sh-VIRMA-1 and sh-HNRNPC-1 had better knockdown effects. Further RT-qPCR detection (Fig. 5F, Fig. S2D) showed that after knocking down VIRMA and HNRNPC, respectively, the expression of TFAP2A was significantly decreased, indicating that VIRMA and HNRNPC can regulate the expression of TFAP2A.

Moreover, EO771 and 4T1.2 cells were further divided into three groups. RT-qPCR results (Fig. 5G, Fig. S2E) showed that compared with the oe-NC + sh-NC group, the expression of VIRMA and TFAP2A was significantly increased, and HNRNPC expression was not significantly changed. In comparison with the one-VIRMA + sh-NC group, VIRMA expression was not significantly changed in the oe-VIRMA + sh-HNRNPC group, and both HNRNPC and TFAP2A expression was significantly decreased, indicating that VIRMA can promote TFAP2A expression and silencing HNRNPC can reverse the promoting effect of VIRMA on TFAP2A expression.

By using Me-RIP to detect the m6A methylation modification level of TFAP2A mRNA in EO771 and 4T1.2 cells, it was found that (Fig. 5H, Fig. S2F) compared with the oe-NC + sh-NC group, the m6A methylation modification level of TFAP2A mRNA was significantly increased in the oe-VIRMA + sh-NC group. However, compared with the oe-VIRMA + sh-NC group, there was no significant change in the m6A methylation modification level of TFAP2A mRNA in the oe-VIRMA + sh-HNRNPC group.

Using VIRMA as an antibody, the PAR-CLIP experiment was conducted to detect the binding of VIRMA to TFAP2A mRNA. The results showed (Fig. 5I, Fig. S2G) that compared with the oe-NC + sh-NC group, the expression level of pulled-down TFAP2A mRNA was significantly increased in the oe-VIRMA + sh-NC group. However, in comparison with the oe-VIRMA + sh-NC group, there was no significant change in the pulled-down TFAP2A mRNA expression level in the oe-VIRMA + sh-HNRNPC group, indicating that VIRMA can promote m6A methylation modification of TFAP2A mRNA and HNRNPC does not affect the m6A modification level of TFAP2A mRNA.

Finally, using HNRNPC as an antibody, the RIP experiment was conducted to detect the recognition of m6A modification of TFAP2A mRNA by HNRNPC. The results showed (Fig. 5J, Fig. S2H) that compared with the oe-NC + sh-NC group, the expression level of pulled-down TFAP2A mRNA was significantly increased in the oe-VIRMA + sh-NC group. Conversely, compared with the oe-VIRMA + sh-NC group, the expression level of pulled-down TFAP2A mRNA was significantly decreased in the oe-VIRMA + sh-HNRNPC group.

**HNRNPC activates the TFAP2A/DDR1 axis by recognizing the m6A modification of TFAP2A mRNA by VIRMA, thereby promoting immune evasion of BC**.

**Fig. 6:**
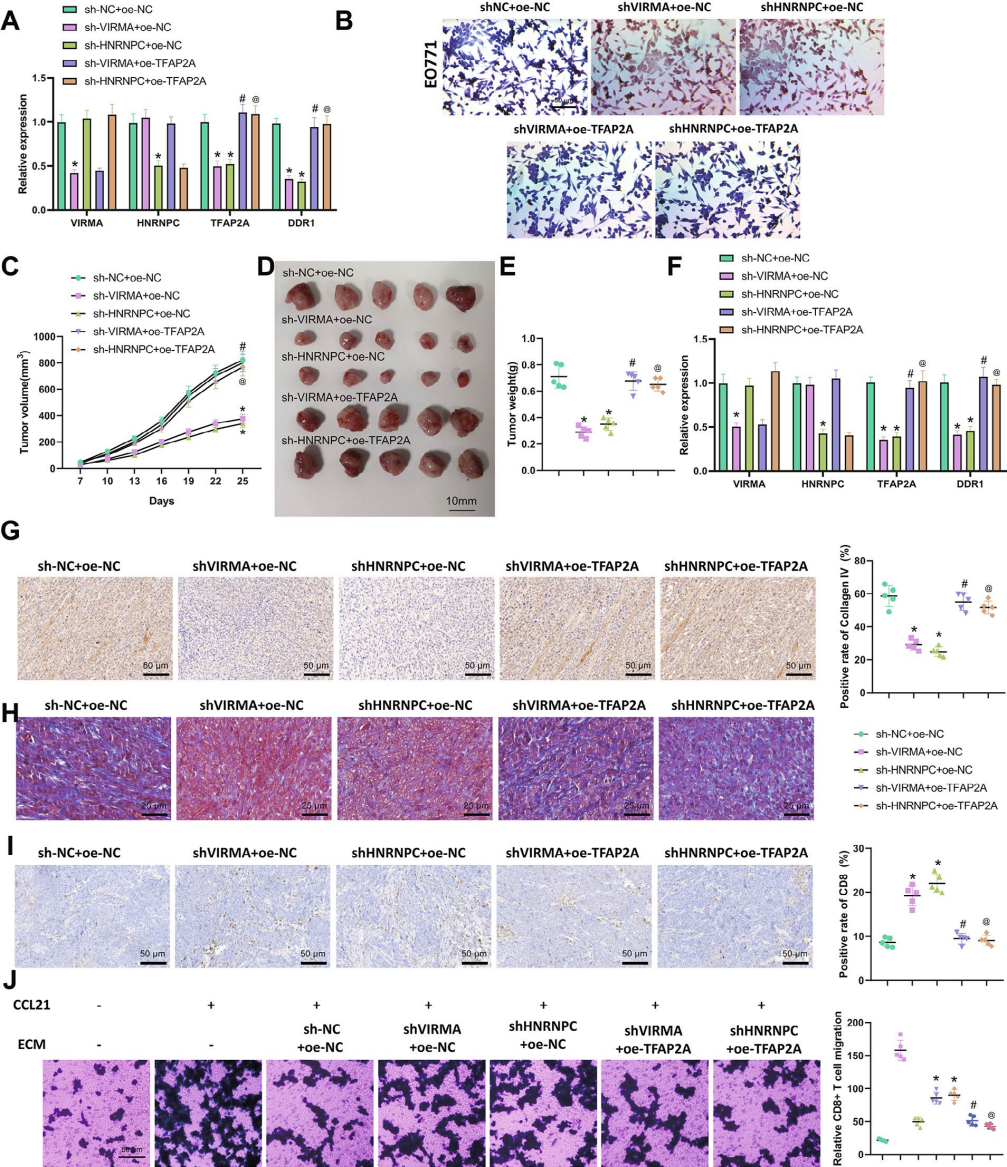
HNRNPC regulates the effect of the TFAP2A/DDR1 axis on collagen fiber arrangement and immune cell infiltration by recognizing the m6A modification of TFAP2A mRNA by VIRMA. Note: (**A**) RT-qPCR detection of the expression levels of VIRMA, HNRNPC, TFAP2A, and DDR1 in each group of EO771 cells; (**B**) Masson staining detection of collagen fiber arrangement in each group of EO771 cells in BC-MPS (×100); (**C**) Tumor volume of each group of breast cancer mice; (**D**) Picture of tumor harvested from each group of breast cancer mice; (**E**) Tumor weight of each group of breast cancer mice; (**F**) RT-qPCR detection of the expression levels of VIRMA, HNRNPC, TFAP2A, and DDR1 in each group of breast cancer mice tumor tissue; (**G**) Immunohistochemistry detection of Collagen IV content in ECM of each group of breast cancer mice tumor tissue (×200); (**H**) Masson staining detection of collagen fiber arrangement in each group of breast cancer mice tumor tissue (×400); (**I**) Immunohistochemistry detection of CD8 positive cell number in each group of breast cancer mice tumor tissue (×200); (**J**) CD8 + T cell migration experiment to detect the number of CD8 + T cell migration in each group of breast cancer mice tumor tissue after decellularized ECM treatment. * indicates P < 0.05 when compared with the two groups. All cell experiments were repeated 3 times with 5 mice in each group

In order to explore the effect of VIRMA and HNRNPC on immune escape in breast cancer through regulation of the TFAP2A/DDR1 axis, we grouped and treated EO771 cells and 4T1.2 cells. RT-qPCR results (Fig. 6A, Fig. S3A) showed that after silencing VIRMA, the expression of VIRMA, TFAP2A, and DDR1 in cells decreased significantly while HNRNPC expression did not change significantly. After silencing HNRNPC, the expression of HNRNPC, TFAP2A, and DDR1 in cells decreased significantly, while VIRMA expression did not change significantly. Overexpression of TFAP2A could promote the expression of TFAP2A and DDR1.

Next, we constructed BC-MPS according to cell grouping, and the Masson staining results (Fig. 6B, Fig. S3B) showed that the collagen fiber distribution in BC-MPS was sparse after silencing VIRMA or HNRNPC. Overexpression of TFAP2A could reverse the collagen fiber arrangement in BC-MPS after silencing VIRMA or HNRNPC, resulting in a higher level of the directional distribution of collagen fibers.

In the in vivo experiment, we grouped and treated EO771-transplanted breast cancer mice. The tumor volume and weight statistics (Fig. 6C-E) showed that the tumor volume and weight decreased significantly after silencing VIRMA or HNRNPC. At the same time, overexpression of TFAP2A could reverse the inhibitory effect of silencing VIRMA or HNRNPC on tumor volume and weight. RT-qPCR results (Fig. 6F) showed that after silencing VIRMA, the expression of VIRMA, TFAP2A, and DDR1 in tumor tissue decreased significantly, while HNRNPC expression did not change significantly. After silencing HNRNPC, the expression of HNRNPC, TFAP2A, and DDR1 in tumor tissue decreased significantly, while VIRMA expression did not change significantly. Overexpression of TFAP2A could promote the expression of TFAP2A and DDR1.

Immunohistochemical and Masson staining results (Fig. 6G-H) showed that the content of Collagen IV in tumor ECM decreased significantly, and the collagen fiber distribution became sparse after silencing VIRMA or HNRNPC. Overexpression of TFAP2A could reverse the content of Collagen IV in tumor ECM and the arrangement of collagen fibers after silencing VIRMA or HNRNPC, resulting in a higher level of the directional distribution of collagen fibers. The immunohistochemical results (Fig. 6I) showed that after silencing VIRMA or HNRNPC, the number of CD8-positive cells in tumor tissue increased significantly, while overexpression of TFAP2A could inhibit the number of CD8-positive cells in tumor tissue. This result shows that VIRMA and HNRNPC can promote the increase of Collagen IV content in ECM and enhance the arrangement of collagen fibers by promoting the TFAP2A/DDR1 axis, thus reducing immune cell infiltration and promoting tumor growth.

The results of the CD8 + T cell migration experiment (Fig. 6J) showed that the number of CD8 + T cell migrations increased significantly after silencing VIRMA or HNRNPC, while overexpression of TFAP2A could inhibit the migration of CD8 + T cells after silencing VIRMA or HNRNPC. This result indicates that VIRMA and HNRNPC can reduce the infiltration of CD8 + T cells in tumors by promoting the TFAP2A/DDR1 axis.

## Discussion

**Fig. 7:**
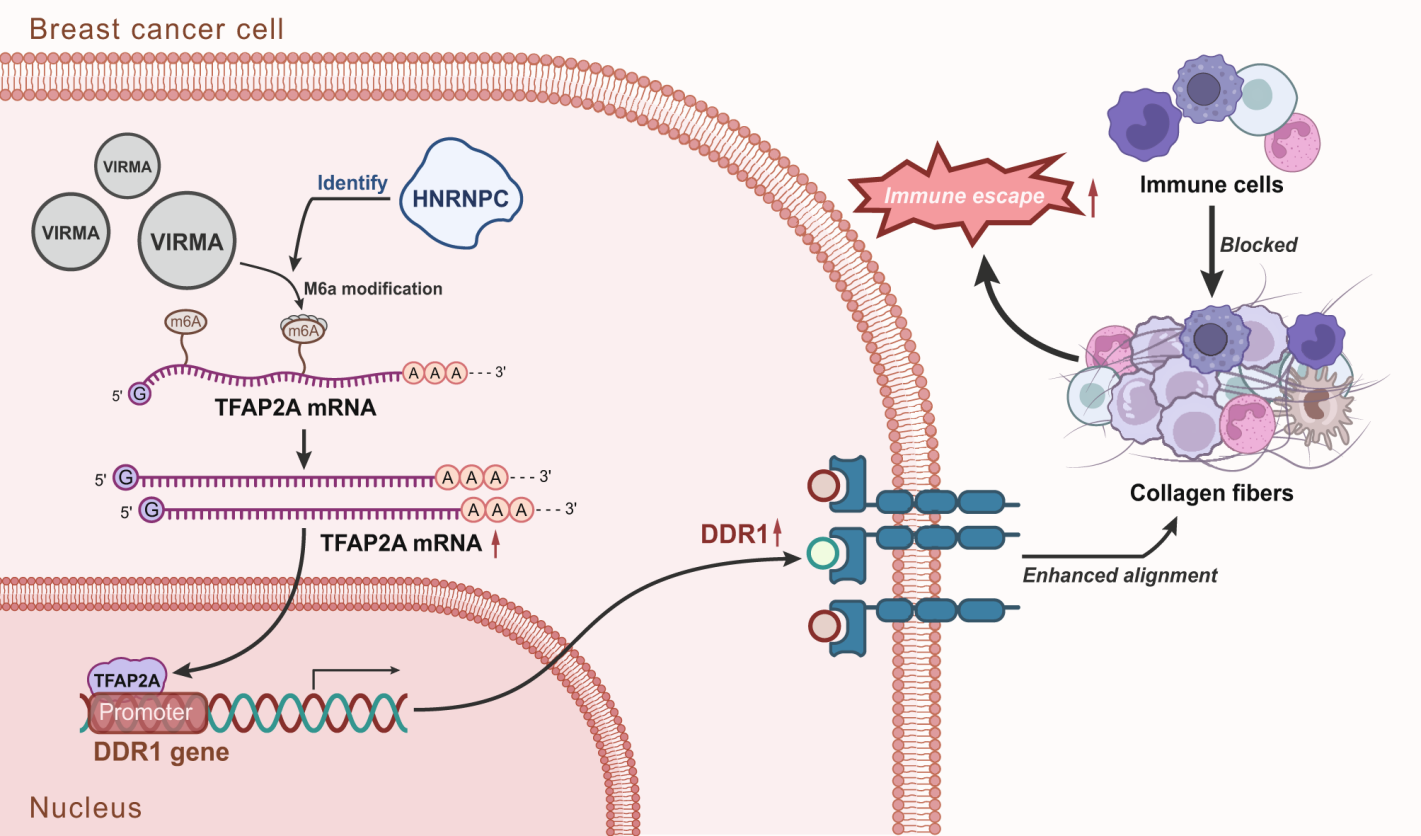
Molecular mechanism diagram of DDR1 regulating type IV collagen fiber synthesis and collagen reorganization in the tumor microenvironment affecting breast cancer immune escape

Collagen fibers surrounding breast ducts may influence BC growth (Zhou et al. 2017; Conklin et al. 2018), leading researchers to examine the underlying mechanism involving collagen fiber alignment in BC. The current results identified a mechanism by which DDR1 promoted collagen IV fiber synthesis, collagen remodeling, and the resultant immune evasion of BC in the tumor microenvironment. Specifically, HNRNPC recognizes the m6A modification of TFAP2A mRNA by VIRMA, consequently increasing TFAP2A expression and promoting DDR1 transcription, thus inducing collagen fiber alignment, inhibiting anti-tumor immune cell infiltration and accelerating immune evasion of BC (Fig. 7).

Initial bioinformatics analysis indicated that DDR1 was highly expressed in BC and inversely correlated to the anti-tumor immune cell infiltration ratio. Consistently, a recent study revealed higher DDR1 expression in BC tissues compared with adjacent normal tissues, and this high expression in 4T1 cells can promote tumor growth in vivo by inhibiting tumor infiltration of T cells, CD4^+^ and CD8^+^ T cells (Zhong et al. 2019). In addition, this study uncovered that DDR1 promoted immune evasion of BC by decreasing CD8^+^ T cell infiltration, enhancing collagen IV content in the ECM, and disrupting collagen fiber alignment. Type IV collagen is a basement membrane protein, and disruption of this ECM structure is the key to cancer invasion (Jansson et al. 2022). As reported, a reduction in the CD8^+^ T cell infiltration is responsible for the induction of immune evasion (Shang et al. 2022). In line with our findings, ablation of DDR1 can disrupt collagen fiber alignment, promote the intratumoral penetration of T cells and retard tumor growth in the mouse models of triple-negative breast cancer, thus preventing immune evasion (Sun et al. 2021). These data highlight the promoting effect of DDR1 on immune evasion of BC, and DDR1 blockade may be a promising target for BC treatment.

Transcription factors are proteins capable of regulating gene activity by activating or repressing gene transcription (Garbuzov and Gursky 2021). In this study, the transcription factor TFAP2A was found to promote DDR1 transcription by binding to the DDR1 promoter region. In addition, TFAP2A could elevate DDR1 expression to reduce BC immune cell infiltration and facilitate collagen fiber alignment. Furthermore, TFAP2A is a target of miR-195-3p, which is positively correlated with the level of immune infiltration of B cells (Lao et al. 2022). In addition, TFAP2A is a target of VEGFA, the expression in patients with renal cell carcinoma shows a positive association with immune cell infiltration, including CD8^+^ T cells and CD4^+^ T cells (Situ et al. 2022). These reports imply the inhibitory role of TFAP2A in tumor immune cell infiltration, which still requires further investigation. In addition, a previous study has unveiled that the knockdown of TFAP2A can significantly decrease the gene expression of collagen IV (Mi et al. 2016).

Mechanistically, the current investigation observed that HNRNPC could augment TFAP2A expression by recognizing the m6A modification of TFAP2A mRNA by VIRMA. VIRMA acts as m6A methyltransferase to promote the m6A modification of mRNAs (Jiang et al. 2021). Moreover, HNRNPC can promote mRNA expression by recognizing m6A modification (Jin et al. 2022). m6A is considered a key regulator of tumors and the immune system and plays an important role in tumor growth and immune evasion (Li et al. 2022). The expression of HNRNPC is negatively correlated with the infiltration of most immune cells in prostate cancer (Wang et al. 2021). Meanwhile, in the tumor microenvironment of renal cell carcinoma, HNRNPC is adversely correlated with infiltration levels of several immune cells, such as natural killer cells, immature dendritic cells, and CD8^+^ T cells (Wu et al. 2022). The aforementioned results and reports serve to illustrate that HNRNPC can activate the TFAP2A/DDR1 axis by recognizing the m6A modification of TFAP2A mRNA by VIRMA to potentiate collagen fiber alignment, and repress anti-tumor immune cell infiltration, ultimately promoting immune evasion of BC.

## Conclusions

In summary, we unraveled a novel HNRNPC/TFAP2A/DDR1 signaling axis that promotes tumor immune evasion by enhancing collagen fiber alignment. The present study not only expands our understanding of m6A-regulated tumor progression from a new perspective but also explains the regulatory mechanism of HNRNPC/TFAP2A/DDR1 in the immune evasion of BC. However, further studies are warranted to investigate the correlation among the HNRNPC, TFAP2A, and DDR1 due to insufficient illustration has been made in their correlation.

## Electronic supplementary material

Below is the link to the electronic supplementary material.

Supplementary Material 1

Supplementary Material 2

Supplementary Material 3

Supplementary Material 4

## Data Availability

The datasets generated and/or analyzed during the current study are available in the manuscript and supplementary materials.

## Abbreviations

BC: Breast cancer
ECM: Extracellular matrix
DDR1: Discoidin domain receptor 1
TFAP2A: Transcription factor AP-2 A
HNRNPC: Heterogeneous nuclear ribonucleoprotein
BC-MPS: BC microphysiological system
ASCs: Adipose tissue-derived stromal cells
