# Supplementary figures and images for: HNRNPC promotes collagen fiber alignment and immune evasion in breast cancer via activation of the VIRMA-mediated TFAP2A/DDR1 axis
Source: Mol Med. 2023 Aug 1;29:103. doi: 10.1186/s10020-023-00696-5 (PMC10394847; doi:10.1186/s10020-023-00696-5)

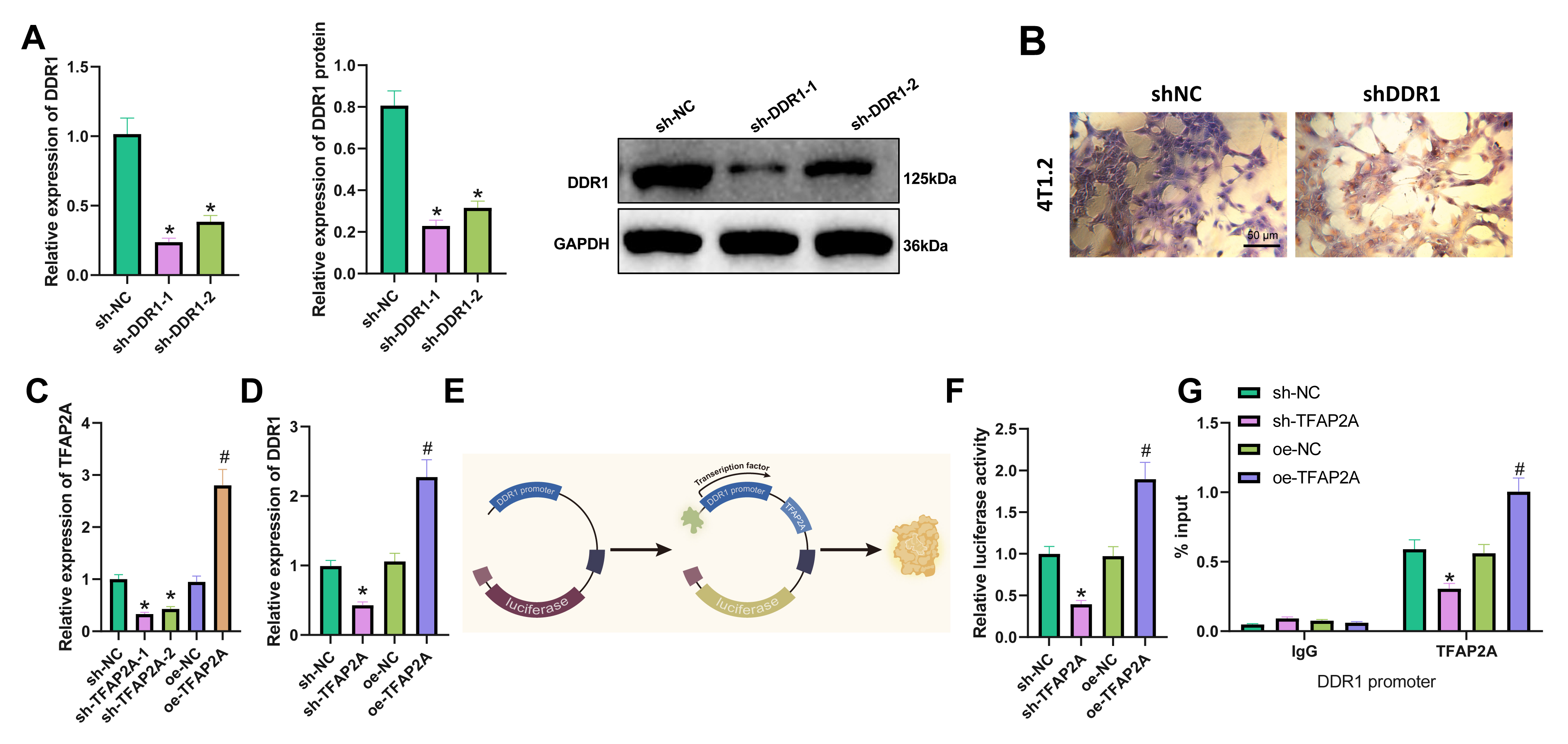

Supplement: Supplementary file 1 — Supplementary Material 1 [file 10020_2023_696_MOESM1_ESM.jpg]
