# Supplementary material for: HNRNPC promotes collagen fiber alignment and immune evasion in breast cancer via activation of the VIRMA-mediated TFAP2A/DDR1 axis
Source: Mol Med. 2023 Aug 1;29:103. doi: 10.1186/s10020-023-00696-5 (PMC10394847; doi:10.1186/s10020-023-00696-5)
Supplement: Supplementary file 4 — Supplementary Material 4 [file 10020_2023_696_MOESM4_ESM.docx]

**Supplementary Table 1. Lentivirus silencing sequence.**

| Target | Sequence (3'-5') |
| --- | --- |
| sh-NC | CTGACTGGGATCAATCGAACT |
| sh-DDR1-1 | GTATTTATCTGAGGCCGTGTA |
| sh-DDR1-2 | CTGGTAGCTGTCAAGATCTTA |
| sh-TFAP2A-1 | CCAATGAGCAAGTGACAAGAA |
| sh-TFAP2A-2 | CCCAGATCAAACTGTAATTAA |
| sh-VIRMA-1 | CCAAGAAATAGTTCGCTCTTT |
| sh-VIRMA-2 | CGGAATATGAAGCAACAAATT |
| sh-HNRNPC-1 | GCCTTCGTTCAGTATGTTAAT |
| sh-HNRNPC-2 | CTGGATGATGATGATAATGAA |

**Supplementary Table 2. RT-qPCR reaction primer sequence.**

| Gene | Sequence (5'-3') |
| --- | --- |
| DDR1 (mouse) | Forward: 5'-GGAACAGGCCCCCGACA-3'  Reverse: 5'-GCTCTGGATCCCTCTGGTCT-3' |
| HNRNPC (mouse) | Forward: 5'-GACTTGTCCTTCTCATCCCCA-3'  Reverse: 5'-ACCTGCCTCAGACTCCATCT-3' |
| VIRMA (mouse) | Forward: 5'-CAGGTTTTTCACACCGCCTG-3'  Reverse: 5'-TAGGGCGGTAACCCGTAGAA-3' |
| TFAP2A (mouse) | Forward: 5'-CAGAGGGGCAAATCCGATCA-3'  Reverse: 5'-AAGTCGGCATTAGGGGTGTG-3' |
| GAPDH (mouse) | Forward: 5'-CCCTTAAGAGGGATGCTGCC-3'  Reverse: 5'-TACGGCCAAATCCGTTCACA-3' |
